# Inequity in postpartum healthcare provision at home and its association with subsequent healthcare expenditure

**DOI:** 10.1093/eurpub/ckz076

**Published:** 2019-04-23

**Authors:** Jacqueline Lagendijk, Eric A P Steegers, Jasper V Been

**Affiliations:** 1 Department of Obstetrics and Gynaecology, Erasmus MC, University Medical Centre Rotterdam, Rotterdam, the Netherlands; 2 Division of Neonatology, Department of Paediatrics, Erasmus MC – Sophia Children’s Hospital, University Medical Centre Rotterdam, Rotterdam, the Netherlands; 3 Department of Public Health, Erasmus MC, University Medical Centre Rotterdam, Rotterdam, the Netherlands

## Abstract

**Background:**

Provision of postpartum care can support new families in adapting to a new situation. We aimed to determine whether various determinants of socioeconomic status (SES) were associated with utilization of postpartum care. In addition, to stress the relevance of increasing postpartum care uptake among low SES-groups, an assessment of the potential (cost-)effectiveness of postpartum care is required.

**Methods:**

National retrospective cohort study using linked routinely collected healthcare data from all registered singleton deliveries (2010–13) in the Netherlands. Small-for-gestational age and preterm babies were excluded. The associations between SES and postpartum care uptake, and between uptake and health care expenditure were studied using multivariable regression analyses.

**Results:**

Of all 569 921 deliveries included, 1.2% did not receive postpartum care. Among women who did receive care, care duration was below the recommended minimum of 24 h in 15.3%. All indicators of low SES were independently associated with a lack in care uptake. Extremes of maternal age, single parenthood and being of non-Dutch origin were associated with reduced uptake independent of SES determinants. No uptake of postpartum care was associated with maternal healthcare expenses in the highest quartile: aOR 1.34 (95% CI 1.10–1.67). Uptake below the recommended amount was associated with higher maternal and infant healthcare expenses: aOR 1.09 (95% CI 1.03–1.18) and aOR 1.20 (95% CI 1.13–1.27), respectively.

**Conclusion:**

Although uptake was generally high, low SES women less often received postpartum care, this being associated with higher subsequent healthcare expenses. Strategies to effectively reduce these substantial inequities in early life are urgently needed.

## Introduction

The postpartum period is a critical transitional period not only for babies but also in the lives of new mothers.^1^ Adequate care provision during this period by skilled maternity care professionals enables an optimal start for the new family. A healthy start following childbirth may be of substantial short and long term benefit for maternal and child wellbeing, and as such has the potential to reduce healthcare associated costs.^2^^,^^3^

The uptake of healthcare overall and the incidence of adverse health outcomes during the postpartum period are closely linked to different determinants of one’s socioeconomic position; persons with a lower socioeconomic position tend to make less use of routine or preventive healthcare,^4^^,^^5^ and have a higher incidence of adverse health outcomes.^3^^,^^6–10^ Although a number of studies examined this relationship, the association between SES and use of postpartum care has not been investigated previously.

**Figure 1:**
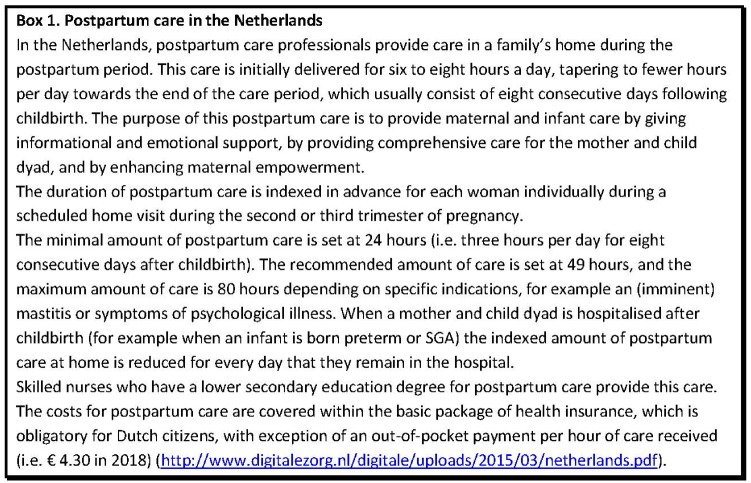
Postpartum care in the Netherlands

The strong position of primary care in the Netherlands, which includes easy access to postpartum care at home during the early postpartum period (figure 1), provides considerable potential to promote equity in maternal and infant health. This study seeks to describe the patterns of utilization of postpartum care services using a national population-based study, assessing: (i) whether different determinants of SES—represented by individual level, household level and area-level indicators—were associated with uptake of postpartum care and (ii) whether any inequalities translated in uptake of care translated into differences in subsequent healthcare expenditures for mother and child in the first year after childbirth, as an estimate of potential (cost-)effectiveness.

## Methods

We conducted a national population-based retrospective cohort study of women living in the Netherlands who delivered a live singleton baby between 1 January 2010 and 31 December 2013. Routinely collected healthcare and claims data were linked at the individual level across various national databases. First, we studied the association between different determinants of low SES and the uptake of postpartum care. Second, we studied the association between the uptake of postpartum care and healthcare expenditures for mother and child in the following year. We used the RECORD statement to guide reporting of our findings (Supplementary file S1).^11^

### Study design and setting

Population-based retrospective cohort study from 1 January 2010 through 31 December 2013 using routinely collected healthcare data from Statistics Netherlands (translated Dutch name: ‘Central Bureau of Statistics’, abbreviation ‘CBS’).

### Participants

All registered pregnancies among women living in the Netherlands who delivered a live singleton baby at 24 or more completed gestational weeks between 2010 and 2013.

### Exposure variables

For the first part of this study, multiple determinants of SES including individual, household and area-level SES indicators constituted the exposures of interest. Disposable household income was used as an individual SES indicator, defined as the sum available from the household income for final consumption and savings (i.e. net income) and divided into quartiles. Mother’s highest educational qualification, based on the International Standard Classification of Education (ISCED) (http://uis.unesco.org/sites/default/files/documents/international-standard-classification-of-education-isced-2011-en.pdf), was considered a second individual SES indicator. Three groups were considered: lower education (pre-primary, primary and lower secondary education), intermediate education (upper secondary education, post-secondary non-tertiary education) and higher education (first stage of tertiary education, second stage of tertiary education).

Home ownership was considered a household indicator of SES and was dichotomized into owner-occupiers and no-owners (i.e. renters and others).

Neighbourhood deprivation was considered an area-level SES indicator and was based on the Neighbourhood Deprivation Index (NDI) formulated by NIVEL in 2012.^12^ Deprivation was defined at an NDI of 5.5% (i.e. 885 000 people).

In the second part, the exposure was the uptake of any postpartum care, and—in a secondary analysis—the uptake of postpartum care above the recommended minimum (i.e. 24 h) among those who did receive postpartum care.

### Covariates

Covariates were selected based on their association with the outcome variables or both the outcome and the exposure variables: maternal age, parity, country of origin, parenthood household status, level of urbanization and small-for-gestational age and preterm babies. Details are presented in Supplementary file S2.

### Outcomes

#### Determinants of low SES and uptake of postpartum care

Uptake of postpartum care was derived from data regarding healthcare expenditures. Expenditures were provided per annum; therefore pregnancies from women who gave birth more than once within 1 year were excluded for all analysis. The amount of postpartum care was calculated by dividing the total postpartum care expenditures within 1 year by the eligible compensation per hour of care, which differed per year.^13^ Uptake of postpartum care was dichotomized into ‘No’, and ‘Yes’ (any amount of postpartum care). The secondary outcome was postpartum care uptake above the minimum (i.e. 24 h), as assessed among all women who did make use of postpartum care. The uptake of the minimum amount of care was dichotomized.

#### Uptake of postpartum care and healthcare expenditures

Annual total healthcare expenditures were obtained separately for mother and child. Quartiles of annual healthcare expenditures were formed for each and dichotomized into ‘low’ (expenses within the first three quartiles) and ‘high’ (expenses in the fourth quartile). Healthcare expenditure data were only available at an aggregated level per annum. We were therefore able to reliably evaluate health care costs in the year post-delivery only among those women delivering close to closing of the year. As such, we pragmatically considered total healthcare costs in the subsequent year following delivery in December a reasonable estimate of healthcare expenditure in the year post-delivery, and excluded deliveries in January to November from these analyses. Healthcare expenditures are subdivided based on a combination of diagnosis and treatment codes enabling us to exclude all healthcare expenses that were labelled as pregnancy-related. In addition, we excluded women with more than one pregnancy during the study period (i.e. 2010–13) because having consecutive pregnancies over a 2-year period could influence healthcare expenditures at the annual level.

### Data sources and linkage

The available data for this study were linked across different national registries by CBS using the unique citizen service number (BSN) or the identification number of the Dutch Population Register (Dutch: A-number). Linkage with this information is feasible in 98–100% of all procedures undertaken by CBS. Details about the individual-level linkage across various routinely collected datasets are presented in Supplementary file S2.

### Potential for bias

The data in this study are based on routinely collected healthcare data. There was a reasonably high proportion of missing values in some registries that could have introduced different biases. We applied multiple imputation using chained equations to account for this missing data in baseline characteristics. Multiple predictor variables were included to inform the multiple imputation process, forming 10 datasets. Results across the sets were combined using Rubin’s Rules.^14^

### Statistical methods

We analysed the two associations under study using logistic regression analysis.

Infants born preterm or SGA and their mothers tend to remain in the hospital during most of the time that the mothers would otherwise be amendable to receiving postpartum care in the home situation (figure 1). Therefore, we excluded deliveries with these outcomes for all analyses because postpartum care uptake would otherwise be underestimated due to prolonged hospital admission.

#### Determinants of low SES and the uptake of postpartum care

The association between various determinants of low SES and postpartum care uptake (first), and uptake above the minimum (second) was analysed. All indicators of SES as exposure variables, and the predefined covariates were included in the analysis to minimize potential confounding.

#### Uptake of postpartum care and healthcare expenditures

The second model analysed the association between postpartum care uptake and healthcare expenditures for mother and child. We accounted for all SES indicators and all covariates included in the first model.

#### Sensitivity analyses

Consecutive pregnancies within the same mother have more characteristics in common than pregnancies between women. To assess whether this dependency of data affected our findings, we reran the model that analyses the first association with additional accounting for clustering at the individual level.

To assess whether the multiple imputed data were biased, we reran the two models on complete cases only.

### Accessibility of protocol and programming code

Upon request all programming codes and the study protocol are available with the principal investigator.

### Details of ethics approval

According to Dutch law, formal ethical assessment of the study protocol was not needed as the study did not involve an intervention and data from CBS are anonymized [based on guidance from the Central Committee on Research Involving Human Subjects (WMO) and the Dutch Personal Data Protection Act].

CBS collects and produces population statistics, referred to as non-public microdata, for all registered Dutch citizens. Under strict conditions, these data are accessible for scientific research. The research board of CBS has reviewed and approved the study protocol (project number 7883).

## Results

### Participants

During the study period, 683 163 deliveries were registered with CBS. After applying the pre-specified exclusion criteria, the final sample included 569 921 deliveries (Supplementary figure S1). For investigation of the association between postpartum care uptake and healthcare expenditures, we additionally excluded deliveries in January through November, and consecutive pregnancies within individual women during the study period. The final sample for this analysis contained 44 458 deliveries (Supplementary figure S1).

### Determinants of low SES and uptake of postpartum care

#### Univariable associations

Table 1 presents the descriptive statistics for the study sample, by uptake of postpartum care. Of all deliveries included, 1.2% did not receive any postpartum care. Data on the uptake of postpartum care were missing for 4.8% of all deliveries. Women who did not use postpartum care were more often: multiparous (67.9% vs. 54.2%), single parents (20.1% vs. 7.7%), born outside the Netherlands (2.9% vs. 0.6%) and they more often lived in deprived neighbourhoods (19.1% vs. 6.8%; table 1). Among women who did receive postpartum care, care duration was below the recommended minimum of 24 h in 15.3% (Supplementary table S1). These deliveries were also associated with indicators of low SES when compared with deliveries with postpartum care uptake above the minimum amount (Supplementary table S1).

**Table 1:** Descriptive statistics of all deliveries by uptake of postpartum care (yes, no, missing)

			Postpartum care uptake					
Total population			Yes		No		Missing	
	*n*=569 921	%	*n*=535 470	%	*n*=6833	%	*n*=27 618	%
Maternal age								
<20	6837	1.2	6231	1.2	237	3.5	369	1.3
20–40	552 753	97.0	519 882	97.1	6325	92.6	26 546	96.1
>40	10 331	1.8	9357	1.7	271	4.0	703	2.5
Parity								
Primiparous	259 330	45.5	245 298	45.8	2196	32.1	11 836	42.9
Multiparous	310 591	54.5	290 172	54.2	4637	67.9	15 782	57.1
Country of origin								
The Netherlands	414 243	72.7	393 408	73.5	2544	37.2	18 291	66.2
Morocco	24 726	4.3	22 920	4.3	980	14.3	826	3.0
Turkey	18 985	3.3	17 989	3.4	515	7.5	481	1.7
Suriname	13 802	2.4	12 864	2.4	372	5.4	566	2.0
Netherlands Antilles	6503	1.1	5864	1.1	189	2.8	450	1.6
Other Non-Western	36 253	6.4	32 077	6.0	1216	17.8	2960	10.7
Other Western	55 409	9.7	50 348	9.4	1017	14.9	4044	14.6
Parenthood status								
Single parent	44 576	7.8	41 130	7.7	1377	20.2	2069	7.5
Two parents	521 140	91.4	491 138	91.7	5184	75.9	24 818	89.9
Other	4197	0.7	3195	0.6	272	4.0	730	2.6
Missing	8	0.0	7	0.0	0	0.0	1	0.0
Urbanized area								
Yes	163 610	28.7	155 696	29.1	1247	18.2	6667	24.1
No	406 311	71.3	379 774	70.9	5586	81.8	20 951	75.9
SES indicators								
Education								
Lower education	74 984	13.2	70 317	13.1	2052	30.0	2615	9.5
Intermediate education	163 305	28.7	156 371	29.2	1438	21.0	5496	19.9
Higher education	197 725	34.7	184 817	34.5	955	14.0	11 953	43.3
Missing	133 907	23.5	123 965	23.2	2388	34.9	7554	27.4
Low-disposable income								
Yes	131 290	23.0	122 207	22.8	3445	50.4	5638	20.4
No	416 580	73.1	393 035	73.4	2993	43.8	20 552	74.4
Missing	22 051	3.9	20 228	3.8	395	5.8	1428	5.2
Home ownership								
No-owners	146 307	25.7	135 846	25.4	3984	58.3	6477	23.5
Owner-occupiers	401 563	70.5	379 396	70.9	2454	35.9	19 713	71.4
Missing	22 051	3.9	20 228	3.8	395	5.8	1428	5.2
Neighbourhood deprivation								
Yes	39 526	6.9	36 248	6.8	1305	19.1	1973	7.1
No	530 395	93.1	499 222	93.2	5528	80.9	25 645	92.9

Values are presented as numbers and percentage.

#### Multivariable associations

All indicators of low SES were consistently and strongly associated with no uptake of postpartum care after mutual adjustment (table 2). Similarly, among mothers who did receive postpartum care, low SES indicators were associated with care uptake below the minimum (table 2). Extremes of maternal age, single parenthood and being of non-Dutch origin were associated with reduced uptake of postpartum care independent of individual and area-level SES.

**Table 2:** Multivariable models of the association between SES indicators and 1) postpartum care uptake and 2) uptake above the minimum (i.e. 24 h)

	Postpartum care uptake (*n* = 569 921)		Uptake above the minimum amount (*n* = 535 470)	
	aOR (95% CI)	*P* value	aOR (95% CI)	*P* value
Covariates				
Maternal age				
<20 years	0.70 (0.61–0.81)	<0.001	0.57 (0.54–0.60)	<0.001
20–40 years (ref)	1		1	
>40 years	0.53 (0.47–0.60)	<0.001	0.78 (0.73–0.82)	<0.001
Parity				
Primiparous (ref)	1		1	
Multiparous	0.59 (0.55–0.62)	<0.001	0.99 (0.98–1.01)	0.601
Parenthood				
Single parent	0.82 (0.76–0.88)	<0.001	0.82 (0.80–0.84)	<0.001
Two parents (ref)	1		1	
Other	0.22 (0.19–0.25)	<0.001	0.40 (0.37–0.43)	<0.001
Country of origin				
The Netherlands (ref)	1		1	
Morocco	0.37 (0.34–0.40)	<0.001	0.23 (0.22–0.24)	<0.001
Turkey	0.44 (0.40–0.49)	<0.001	0.26 (0.25–0.27)	<0.001
Suriname	0.46 (0.41–0.51)	<0.001	0.29 (0.28–0.30)	<0.001
Net. Antilles	0.50 (0.43–0.58)	<0.001	0.32 (0.30–0.34)	<0.001
Other Non-Western	0.40 (0.37–0.43)	<0.001	0.22 (0.22–0.23)	<0.001
Other Western	0.41 (0.41–0.48)	<0.001	0.41 (0.40–0.42)	<0.001
SES indicators				
Individual				
Education				
Lower education	0.62 (0.57–0.66)	<0.001	0.65 (0.64–0.67)	<0.001
Inter. education (ref)	1		1	
Higher education	1.21 (1.12–1.32)	<0.001	1.39 (1.36–1.42)	<0.001
Low-disposable income				
Yes	0.72 (0.68–0.77)	<0.001	0.69 (0.68–0.71)	<0.001
Household				
Home ownership				
No-owners	0.55 (0.52–0.59)	<0.001	0.51 (0.50–0.52)	<0.001
Owner-occupiers (ref)	1		1	
Area-level				
Neighbourhood deprivation				
Yes	0.80 (0.75–0.85)	<0.001	0.79 (0.77–0.81)	<0.001

Presented are adjusted odds ratios (aOR) and their 95% confidence intervals (95%CI). All p-values are two-sided. Results for the uptake of care and the minimum uptake of care are presented separately.

#### Sensitivity analyses

Consistent results were obtained in all sensitivity analyses for robustness checks, including those accounting for clustering of pregnancies at the individual level, and those analysing complete cases only (Supplementary table S2).

### Uptake of postpartum care and healthcare expenditures

#### Univariable associations

Descriptive statistics for the subgroup of 44 458 deliveries in December, were similar to those of all deliveries (Supplementary table S3). The prevalence of low SES indicators increased steadily across the four quartiles of maternal healthcare expenditure, with the highest quartile having the highest prevalence of low SES indicators: lowest educational level 23.0% in the highest quartile vs. 17.3% across the other quartiles, low disposable income 30.0% vs. 25.2%, no home-ownership 34.4% vs. 27.7% and living in a deprived neighbourhood 9.0% vs. 7.1% (Supplementary table S3). This tendency was not seen across the four quartiles of infant healthcare expenditure, were the prevalence of low maternal SES indicators in the highest quartile was comparable with the prevalence across the other quartiles (data not presented). The percentage of women who did not receive postpartum care was highest in the fourth quartile of maternal healthcare expenses (2.1% in the highest quartile vs. 1.2% across the other quartiles; Supplementary table S3).

#### Multivariable associations

Not receiving postpartum care, or having postpartum care uptake below the minimum, was associated with a significantly higher odds of having maternal healthcare expenditure within the highest quartile in the year following child birth: aOR 1.34; 95% CI 1.10–1.67; *P* 0.004, and aOR 1.09; 95% CI 1.03–1.18; *P* 0.005, respectively (table 3). Deliveries followed by postpartum care uptake below the minimum were in addition associated with infant healthcare expenditure within the highest quartile in the first year after birth (aOR 1.20; 95% CI 1.13–1.27; *P* < 0.001) (table 3).

**Table 3:** Multivariable model of the association between the uptake of postpartum care and the lack in uptake and maternal and infant healthcare expenses within the highest quartile

	Maternal health care expenditures highest quartile		Infant health care expenditures highest quartile	
	aOR (95% CI)	*P* value	aOR (95% CI)	*P* value
Primary analyses				
Maternity care uptake (*n*=44 458)				
No	1.34 (1.10–1.67)	0.004	1.12 (0.94–1.35)	0.205
Yes (ref)	1		1	
Maternity care >24 h (*n*=41 583)				
No	1.09 (1.03–1.18)	0.005	1.20 (1.13–1.27)	<0.001
Yes (ref)	1		1	

Presented are adjusted odds ratios (aOR) and their 95% confidence intervals (95%CI). All p-values are two-sided. Results are presented separately for maternal and infant healthcare expenditures, adjusted for maternal age, parity, country of origin, parenthood status, and all indicators of SES (i.e. educational level, disposable income, home ownership, and neighborhood poverty)

#### Sensitivity analyses

The association between no uptake of postpartum care and maternal healthcare expenses during the first year after childbirth was consistent in the sensitivity analysis using complete cases only [aOR 1.54 (95% CI 1.23–1.92; *P* < 0.001)].

## Discussion

Using a national linked dataset of over half a million singleton pregnancies, we found that all indicators of low SES were associated with no uptake of postpartum care and with uptake of care below the recommended minimum. This lack of postpartum care uptake was associated with higher healthcare expenses in the first year after childbirth. For the first time, we demonstrate that postpartum care may be a cost-effective tool but is least provided to those who are most likely to benefit from it.

Strengths of this study include the very large and nationally representative sample and the use of a unique individual-level linkage across various routinely collected datasets of relevant medical and socioeconomic data. The relationship between low SES and lack of uptake of postpartum care was highly consistent across the various SES indicators. Although at the population level the proportion of women not receiving postpartum care is very small, we have shown that these women represent a marginalized group and may therefore benefit from efforts to improve their care.

In addition, associations between low SES and postpartum care uptake as well as between postpartum care uptake and subsequent healthcare expenditure showed a dose-response association. The largest differences were present between women who did not receive postpartum care and those who received care above the minimum amount. The findings of both analyses were furthermore highly robust in sensitivity analyses. In the absence of major changes to the system used for indicating the amount of postpartum care and of the health care insurance system in the Netherlands, the data used in this study (from 2010 to 2013) may be considered generalizable to the current day.

Our study also has limitations. First, some of the national registries from Statistics Netherlands have a reasonably high percentage of missing values. For example, the percentage of missing values on a woman’s highest educational qualification was as high as 30%. Upward educational-attainment biases could have influenced the registered data. To minimize bias within the imputed data, we had all SES indicators and outcome variables inform the imputation process. Sensitivity analyses on complete cases only showed similar results to the main analyses, supporting validity of the imputation and robustness of the findings. Second, we lacked information on medical conditions of women and infants. Having a medical condition that requires inpatient treatment could directly affect the uptake of postpartum care, as this care is provided only in the primary care setting (i.e. at home or in a primary care birth center). A third limitation is that the provided postpartum care is expressed in total expense rather than days of care received, making derivation necessary. In addition, we did not have information on the number of days spent in the hospital prior to receiving postpartum care, which may have biased our findings. Somewhat related to this point is that healthcare expenditures were only available at the annual level. We pragmatically addressed this by only assessing deliveries in December when exploring the association between postpartum care uptake and healthcare expenditure. Although this substantially reduced sample size, statistically significant and clinically relevant associations were still observed.

Our findings stress the need to further explore how equity in care uptake may be promoted. Obstetric healthcare providers should include the social determinants of health in their medical records, and in the referral to postpartum care organizations.^2^^,^^15–17^ Provision of postpartum care should be tailored according to these determinants to reach poor and other marginalized subpopulations.^18^^,^^19^ When striving to reduce inequalities in uptake of postpartum care additional determinants, besides those related to a person’s SES, should be considered. For example, our results showed that immigrant populations were less likely to receive postpartum care, even when accounting for SES indicators (table 2). This suggests that interventions targeting high-risk groups to increase postpartum care uptake should consider ethnic background in addition to SES-related factors. Cultural factors are likely to explain at least part of this inequity, but this requires further study. Mixed-methods research is needed to assess the facilitators and barriers to receiving postpartum care among low-SES women and those with an immigrant background.

Our results are in line with those observed in other reports; there is a consistent inequity in primary care provision, where more care is provided to the well-off, who need it less, than to the more disadvantaged.^2^^,^^4^^,^^5^^,^^16–18^^,^^20^ In the Netherlands, a possible barrier to postpartum care uptake is the additional co-payment required for each hour of care. To ensure equitable, universal coverage, policy-makers and health insurers should consider waiving this co-payment, particularly for low SES groups. Furthermore, our study provides evidence to suggest that postpartum care may help reduce subsequent healthcare expenditure, providing an additional incentive for stakeholders to invest in increasing the uptake of care. There is a need to further assess whether explicit resource allocation and priority setting to those in greatest need, perhaps in conjunction with approaches to reduce unnecessary care provision resulting in the over-payment in other sectors may help improve cost-effectiveness of postpartum care provision.

Given the observational nature of this study it is important that findings are reproduced in other settings or using different methodological approaches (i.e. prospective or randomized studies). Future research should focus on further analysing the (cost-)effectiveness of postpartum care; not only its effectiveness in achieving equity in care provision but also its ability to prevent illness and associated healthcare needs. An in-depth economic evaluation taking into account all expenses made by postpartum care organizations and the potential benefits (e.g. health benefits or value-based health measures) gained by mothers and their children could strengthen a renewed allocation for care provision.

## Supplementary Material

ckz076_Supplementary_Data
