# Supplementary material for: Inequity in postpartum healthcare provision at home and its association with subsequent healthcare expenditure
Source: Eur J Public Health. 2019 Apr 23;29(5):849–55. doi: 10.1093/eurpub/ckz076 (PMC6761843; doi:10.1093/eurpub/ckz076)
Supplement: ckz076_Supplementary_Data [file ckz076_supplementary_data.zip › ckz076-Suppl_data/Supplementary_Data2.docx]

**SUPPLEMENTARY FILE 2:

Definition and categorisation of all exposure variables and covariates**
Covariates

Maternal age at delivery was calculated as date of delivery minus mother’s date of birth and categorized in three categories (<20 years, 20-40 years, >40 years of age). Parity was defined as the number of previous deliveries of each mother and dichotomized into primiparous and multiparous women. Country of origin was defined by birthplace of the woman according to the Dutch Population Register and categorized according to the largest subgroups into: the Netherlands, Morocco, Turkey, Suriname, Netherlands Antilles, other non-Western, and other Western. Parenthood household status was categorized into single parent household, two parent household, and other, the latter including institutionalized women and households not further specified. The level of urbanization, based on address density within a radius of one kilometre around an address, divided by the area of ​​the circle, was dichotomized into ‘rural’ (<1000 addresses), and ‘urban’ (≥1000 addresses).
In addition, two perinatal characteristics were defined: preterm delivery defined as a delivery before 37 completed weeks of gestation, and delivery of a small for gestational age baby (SGA), defined as a birth weight below the 10^th^ centile for parity, gestational age, and gender ^1^.

**Data sources and linkage**

The available data for this study was linked across different national registries by CBS using the citizen service number (BSN) or the identification number of the Dutch Population Register (Dutch: A-number). Alternatively, a person’s gender, date of birth, postal code, and year of address registration is used for linkage.
Pregnancy data was obtained from the Dutch medical birth registry (‘Perined’), which includes data of pregnancies, including stillbirths, of 22 or more completed gestational weeks, of all women registered in the Dutch Register of Population. Approximately 97% of all births in The Netherlands are registered with Perined ^2^.
We obtained information on maternal and neonatal deaths by using the Cause of Death register, which includes information on all deaths in the Netherlands (<https://www.cbs.nl/-/media/cbs%20op%20maat/microdatabestanden/documents/2016/30/do.pdf>). Diagnoses and causes of death are coded according to the Dutch version of the International Classification of Diseases, tenth Revision (ICD-10).
Data regarding participants’ educational level was collected from the central registry for application into tertiary education (Dutch abbreviation ‘CRIHO’) and the registry for exam results for secondary education (Dutch abbreviation ‘ERR’). To complement the data of these two registries, an annual survey assessing a person’s educational skills is conducted by Statistics Netherlands (<https://www.cbs.nl/-/media/cbs%20op%20maat/microdatabestanden/documents/2016/37/hoogsteopltab.pdf>). The educational data covers approximately 70% of the adult Dutch population.
Income data was collected from the Dutch Tax Service registry (<https://www.cbs.nl/-/media/cbs%20op%20maat/microdatabestanden/documents/2016/51/integraal%20persoonlijk%20inkomen.pdf>). Data regarding a woman’s residency based on postal codes, her parental status, and home ownership was obtained from the Dutch Population Register (<https://www.cbs.nl/-/media/cbs%20op%20maat/microdatabestanden/documents/2017/16/gbaadresobjectbus.pdf>). Registering oneself in the Dutch Population Register is obligatory, except for North Atlantic Treaty Organization (NATO)-soldiers, diplomats, and residents who remain in the Netherlands for less than four months within a period of six months (<https://www.government.nl/binaries/government/documents/leaflets/2017/01/19/brochure-brp-engelstalig/Brochure+BRP+-+Engelstalig+-+def+versie+voor+publicatie+lowres.pdf> ). An important group missing in the Dutch population Register are illegal immigrants (estimated percentage of the total Dutch population in 2001: 0.29-0.72%)^3^. The Dutch index of deprivation (abbreviation in Dutch language: NIVEL) is an area-based measure of deprivation that ranks small geographical areas (i.e. based on postal codes) on the basis of multiple aspects of deprivation identified in administrative data ^4^. This index was used to identify deprived areas. We assessed the level of urbanization based on a woman’s postal code. Postal codes obtained from Dutch Population Register were linked with the date of delivery to obtain precise information with regard to maternal living condition at the time of childbirth. The level of urbanization was based on address density per area.
Vektis is a private organization that collects costs made by everyone with a basic package of health insurance at annual level. Data regarding health expenditures covers approximately 95% of the Dutch population (<https://www.cbs.nl/-/media/cbs%20op%20maat/microdatabestanden/documents/2017/27/zvwzorgkostentab.pdf>). Health care expenditures are subdivided based on a combination of diagnosis and treatment. Information regarding the uptake of maternity care is based on a subdivision of expenses made during pregnancy and the postpartum period.
Individual-level data on health care expenditures were available aggregated per year from 2011-2014. Health care costs can be invoiced with a delay, to obtain an accurate number for expenditures per annum; expenses were therefore calculated with data across seven quarters of a year (i.e. four quarters of the actual year and the first three quarters of the subsequent year) according to the standardized method of Statistics Netherlands. For women delivering in December the health care costs from the following year were considered a reasonable estimate of the costs made in the first year after childbirth.

**References**

1 Visser GH, Eilers PH, Elferink-Stinkens PM, Merkus HM, Wit JM. New Dutch reference curves for birthweight by gestational age. Early Hum Dev 2009;85:737-744.

2 Netherlands S. Documentatierapport Perinatale Registratie Nederland (PRN). <https://wwwcbsnl/en-gb/our-services/customised-services-microdata/microdata-conducting-your-own-research>.

3 Hoogteijling EMJ. Raming van het aantal niet in de GBA geregistreerden. <https://wwwcbsnl/nl-nl/achtergrond/2002/16/raming-van-het-aantal-niet-in-de-gba-geregistreerden> 2002;April 2002.

4 Devillé W WT. Herijking stedelijke achterstandsgebieden. <https://wwwnivelnl/en/ethnic-and-socio-economic-differences-in-health-and-illness> 2012.
