# Supplementary material for: Inequity in postpartum healthcare provision at home and its association with subsequent healthcare expenditure
Source: Eur J Public Health. 2019 Apr 23;29(5):849–55. doi: 10.1093/eurpub/ckz076 (PMC6761843; doi:10.1093/eurpub/ckz076)
Supplement: ckz076_Supplementary_Data [file ckz076_supplementary_data.zip › ckz076-Suppl_data/Supplementary_Data3.docx]

Supplementary table 1: Descriptive statistics by uptake of postpartum care above the recommended minimum.

|  |  | | Postpartum care uptake above minimum amount | | | | |
| --- | --- | --- | --- | --- | --- | --- | --- |
| Total population | | |  | **Yes** |  | **No** |  |
|  | | n=535 470 | % | n=453 762 | % | n=81 708 | % |
| Maternal age | |  |  |  |  |  |  |
| <20 | | 6231 | 1,2 | 3509 | 0,8 | 2722 | 3,3 |
| 20-40 | | 519 882 | 97,1 | 442 757 | 97,6 | 77 125 | 94,4 |
| >40 | | 9357 | 1,7 | 7496 | 1,7 | 1861 | 2,3 |
| Parity | |  |  |  |  |  |  |
| Primiparous | | 245 298 | 45,8 | 208 914 | 46,0 | 36 384 | 44,5 |
| Multiparous | | 290 172 | 54,2 | 244 848 | 54,0 | 45 324 | 55,5 |
| Country of origin | |  |  |  |  |  |  |
| the Netherlands | | 393 408 | 73.5 | 361 802 | 79.7 | 31 606 | 38.7 |
| Morocco | | 22 920 | 4.3 | 12 235 | 2.7 | 10 685 | 13.0 |
| Turkey | | 17 989 | 3.4 | 11 034 | 2.4 | 6 955 | 8.5 |
| Suriname | | 12 864 | 2.4 | 8 146 | 1.8 | 4 718 | 5.8 |
| Netherlands Antilles | | 5 864 | 1.1 | 3 534 | 0.8 | 2 330 | 2.9 |
| Other Non-Western | | 32 077 | 6.0 | 18 040 | 4.0 | 14 037 | 17.2 |
| Other Western | | 50 348 | 9.4 | 38 971 | 8.6 | 11 377 | 13.9 |
| Parenthood status | |  |  |  |  |  |  |
| Single parent | | 41 130 | 7,7 | 26 148 | 5,8 | 14 982 | 18,3 |
| Two parents | | 491 138 | 91,7 | 425 631 | 93,8 | 65 507 | 80,2 |
| Other | | 3195 | 0,6 | 1978 | 0,4 | 1217 | 1,5 |
| Missing | | 7 | 0,0 | 5 | 0,0 | 2 | 0,0 |
| Urbanized area | |  |  |  |  |  |  |
| Yes | | 155 696 | 29,1 | 143 085 | 31,5 | 12 611 | 15,4 |
| No | | 379 774 | 70,9 | 310 677 | 68,5 | 69 097 | 84,6 |
| SES indicators | |  |  |  |  |  |  |
| Education | |  |  |  |  |  |  |
| Lower education | | 70 317 | 13,1 | 46 009 | 10,1 | 24 308 | 29,7 |
| Intermediate education | | 156 371 | 29,2 | 134 850 | 29,7 | 21 521 | 26,3 |
| Higher education | | 184 817 | 34,5 | 172 643 | 38,0 | 12 174 | 14,9 |
| Missing | | 123 965 | 23,2 | 100 260 | 22,1 | 23 705 | 29,0 |
| Low disposable income | |  |  |  |  |  |  |
| Yes | | 122 207 | 22,8 | 84 011 | 18,5 | 38 196 | 46,7 |
| No | | 393 035 | 73,4 | 354 035 | 78,0 | 39 000 | 47,7 |
| Missing | | 20 228 | 3,8 | 15 716 | 3,5 | 4512 | 5,5 |
| Home ownership | |  |  |  |  |  |  |
| No-owners | | 135 846 | 25,4 | 90 553 | 20,0 | 45 293 | 55,4 |
| Owner-occupiers | | 379 396 | 70,9 | 347 493 | 76,6 | 31 903 | 39,0 |
| Missing | | 20 228 | 3,8 | 15 716 | 3,5 | 4512 | 5,5 |
| Neighbourhood deprivation | | |  |  |  |  |  |
| Yes | | 36 248 | 6,8 | 22 004 | 4,8 | 14 244 | 17,4 |
| No | | 499 222 | 93,2 | 431 758 | 95,2 | 67 464 | 82,6 |

Values are presented as numbers and percentage.

|  |  | Addition of clustering on individual level | | | | Complete cases | | | |
| --- | --- | --- | --- | --- | --- | --- | --- | --- | --- |
|  |  | **Maternity care uptake (n=569,921)** | | **Minimum uptake of care (n=535,470)** | | **Maternity care uptake (n=399,529)** | | **Minimum uptake of care (n=395,329)** | |
| *Socioeconomic indicators* | |  |  |  |  |  |  |  |  |
| *Individual* |  | aOR (95% CI) | p-value | aOR (95% CI) | p-value | aOR (95% CI) | p-value | aOR (95% CI) | p-value |
|  | **Education** |  |  |  |  |  |  |  |  |
|  | Lower education | 0.61 (0.57-0.66) | <0.001 | 0.65 (0.64-0.67) | <0.001 | 0.62 (0.57-0.67) | <0.001 | 0.57 (0.56-0.59) | <0.001 |
|  | Intermediate education (ref) | 1 |  | 1 |  | 1 |  | 1 |  |
|  | Higher education | 1.21 (1.11-1.32) | <0.001 | 1.39 (1.36-1.42) | <0.001 | 1.26 (1.16-1.38) | <0.001 | 1.59 (1.55-1.63) | <0.001 |
|  | **Low disposable income** |  |  |  |  |  |  |  |  |
|  | Yes | 0.72 (0.68-0.77) | <0.001 | 0.69 (0.67-0.71) | <0.001 | 0.68 (0.63-0.74) | <0.001 | 0.73 (0.71-0.74) | <0.001 |
|  | No (ref) | 1 |  |  |  | 1 |  |  |  |
| *Household* |  |  |  |  |  |  |  |  |  |
|  | **Home ownership** |  |  |  |  |  |  |  |  |
|  | No-owners | 0.56 (0.52-0.60) | <0.001 | 0.51 (0.50-0.52) | <0.001 | 0.58 (0.53-0.63) | <0.001 | 0.51 (0.49-0.52) | <0.001 |
|  | Owner-occupiers (ref) | 1 |  | 1 |  | 1 |  | 1 |  |
| *Area-level* |  |  |  |  |  |  |  |  |  |
|  | **Neighbourhood deprivation** |  |  |  |  |  |  |  |  |
|  | Yes | 0.80 (0.75-0.86) | <0.001 | 0.78 (0.77-0.81) | <0.001 | 0.80 (0.73-0.87) | <0.001 | 0.77 (0.74-0.79) | <0.001 |
|  | No (ref) | 1 |  | 1 |  | 1 |  | 1 |  |

Supplementary table 2: sensitivity analyses of the association between SES indicators and maternity care uptake (first) and uptake above the recommended minimum (second) (model I).

Presented are adjusted odds ratios (aOR) and their 95% confidence intervals (95%CI). All p-values are two-sided. Results for the uptake of care and the minimum uptake of care are presented separately. Adjusted for maternal age, parity, country of origin, parental status, and urbanised area.

|  | Total population | | Quartiles of maternal health care expenditures | | | | | | | |
| --- | --- | --- | --- | --- | --- | --- | --- | --- | --- | --- |
|  | **n=44 458** | | **Q1 (n=10 551)** | | **Q2 (n=12 079)** | | **Q3 (n=12 278)** | | **Q4 (n=9550)** | |
| Maternal age (years) |  |  |  |  |  |  |  |  |  |  |
| <20 | 585 | 1,3 | 78 | 0,7 | 156 | 1,3 | 198 | 1,6 | 153 | 1,6 |
| 20-40 | 43 060 | 96,9 | 10 295 | 97,6 | 11 718 | 97,0 | 11 848 | 96,5 | 9199 | 96,3 |
| >40 | 813 | 1,8 | 178 | 1,7 | 205 | 1,7 | 232 | 1,9 | 198 | 2,1 |
| Parity |  |  |  |  |  |  |  |  |  |  |
| Primiparous | 21 583 | 48,5 | 5159 | 48,9 | 5968 | 49,4 | 5884 | 47,9 | 4572 | 47,9 |
| Multiparous | 22 875 | 51,5 | 5392 | 51,1 | 6111 | 50,6 | 6394 | 52,1 | 4978 | 52,1 |
| Country of origin |  |  |  |  |  |  |  |  |  |  |
| the Netherlands | 31 724 | 71.4 | 7 884 | 74.7 | 8 704 | 72.1 | 8 640 | 70.4 | 6 496 | 68.0 |
| Morocco | 2 044 | 4.6 | 296 | 2.8 | 537 | 4.5 | 643 | 5.2 | 568 | 6.0 |
| Turkey | 1 563 | 3.5 | 285 | 2.7 | 408 | 3.4 | 473 | 3.9 | 397 | 4.2 |
| Suriname | 1 188 | 2.7 | 204 | 1.9 | 315 | 2.6 | 370 | 3.0 | 299 | 3.1 |
| Netherlands Antilles | 512 | 1.1 | 98 | 0.9 | 145 | 1.2 | 161 | 1.3 | 108 | 1.1 |
| Other Non-Western | 2 938 | 6.6 | 613 | 5.8 | 756 | 6.3 | 863 | 7.0 | 706 | 7.4 |
| Other Western | 4 489 | 10.1 | 1 171 | 11.1 | 1 214 | 10.1 | 1 128 | 9.2 | 976 | 10.2 |
| Parenthood status |  |  |  |  |  |  |  |  |  |  |
| Single parent | 3851 | 8,7 | 648 | 6,1 | 904 | 7,5 | 1207 | 9,8 | 1092 | 11,4 |
| Two parents | 40 245 | 90,5 | 9836 | 93,2 | 11 100 | 91,9 | 10 960 | 89,3 | 8349 | 87,4 |
| Other | 362 | 0,8 | 67 | 0,6 | 75 | 0,6 | 111 | 0,9 | 109 | 1,1 |
| Urbanized area |  |  |  |  |  |  |  |  |  |  |
| Yes | 12 651 | 28,5 | 3497 | 33,1 | 3454 | 28,6 | 3274 | 26,7 | 2426 | 25,4 |
| No | 31 807 | 71,5 | 7054 | 66,9 | 8625 | 71,4 | 9004 | 73,3 | 7124 | 74,6 |
| Education |  |  |  |  |  |  |  |  |  |  |
| Lower education | 6382 | 18,6 | 1111 | 13,7 | 1653 | 17,5 | 1938 | 20,3 | 1680 | 23,0 |
| Intermediate education | 13 077 | 38,0 | 2976 | 36,7 | 3522 | 37,4 | 3770 | 39,5 | 2809 | 38,4 |
| Higher education | 14 922 | 43,4 | 4022 | 49,6 | 4245 | 45,1 | 3825 | 40,1 | 2830 | 38,7 |
| Missing | 10 077 | 22,7 | 2442 | 23,1 | 2659 | 22,0 | 2745 | 22,4 | 2231 | 23,4 |
| Low disposable income |  |  |  |  |  |  |  |  |  |  |
| Yes | 11 194 | 26,3 | 2323 | 22,9 | 2828 | 24,5 | 3299 | 28,0 | 2744 | 30,0 |
| No | 31 435 | 73,7 | 7810 | 77,1 | 8721 | 75,5 | 8497 | 72,0 | 6407 | 70,0 |
| Missing | 1829 | 4,1 | 418 | 4,0 | 530 | 4,4 | 482 | 3,9 | 399 | 4,2 |
| Home ownership |  |  |  |  |  |  |  |  |  |  |
| No-owners | 12 417 | 29,1 | 2514 | 24,8 | 3129 | 27,1 | 3626 | 30,7 | 3148 | 34,4 |
| Owner-occupiers | 30 212 | 70,9 | 7619 | 75,2 | 8420 | 72,9 | 8170 | 69,3 | 6003 | 65,6 |
| Missing | 1829 | 4,1 | 418 | 4,0 | 530 | 4,4 | 482 | 3,9 | 399 | 4,2 |
| Neighbourhood deprivation |  |  |  |  |  |  |  |  |  |  |
| Yes | 3327 | 7,5 | 590 | 5,6 | 868 | 7,2 | 1005 | 8,2 | 864 | 9,0 |
| No | 41 131 | 92,5 | 9961 | 94,4 | 11 211 | 92,8 | 11 273 | 91,8 | 8686 | 91,0 |
| Maternity care uptake |  |  |  |  |  |  |  |  |  |  |
| No | 582 | 1,4 | 138 | 1,3 | 140 | 1,2 | 145 | 1,2 | 159 | 2,1 |
| Yes | 41 583 | 98,6 | 10 297 | 98,7 | 11 832 | 98,8 | 12 008 | 98,8 | 7448 | 97,9 |
| Missing | 2293 | 5,2 | 116 | 1,1 | 109 | 0,9 | 125 | 1,0 | 1943 | 20,3 |

Values are presented as numbers and percentage.

Supplementary table 3: Descriptive statistics of all deliveries in December (model II) by quartiles of maternal health care expenditures.
