# Supplementary figures and images for: Inequity in postpartum healthcare provision at home and its association with subsequent healthcare expenditure
Source: Eur J Public Health. 2019 Apr 23;29(5):849–55. doi: 10.1093/eurpub/ckz076 (PMC6761843; doi:10.1093/eurpub/ckz076)

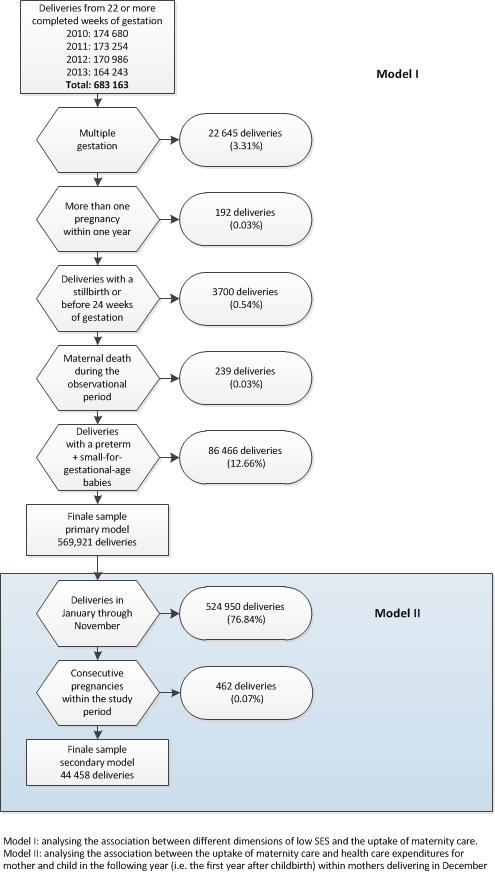

Supplement: ckz076_Supplementary_Data [file ckz076_supplementary_data.zip › ckz076-Suppl_data/Supplementary_Fig.jpg]
